# Overexpression of *Rosea1* From Snapdragon Enhances Anthocyanin Accumulation and Abiotic Stress Tolerance in Transgenic Tobacco

**DOI:** 10.3389/fpls.2018.01070

**Published:** 2018-08-15

**Authors:** Aung H. Naing, Trinh N. Ai, Ki B. Lim, In J. Lee, Chang K. Kim

**Affiliations:** ^1^Department of Horticultural Science, Kyungpook National University, Daegu, South Korea; ^2^School of Agriculture and Aquaculture, Tra Vinh University, Tra Vinh, Vietnam; ^3^School of Applied Biosciences, Kyungpook National University, Daegu, South Korea

**Keywords:** anthocyanin, cold stress, drought stress, gene expression, reactive oxygen species scavenging, phylogenetic analysis, stress tolerance

## Abstract

The co-expression of *Rosea1* (*Ros1*) and *Delila* (*Del*) regulates anthocyanin levels in snapdragon flowers, as well as in tomato, petunia, and tobacco. However, there is little information on how *Ros1* expression alone controls anthocyanin regulation and whether it is involved in the mechanism that leads to abiotic stress tolerance. In the present study, tobacco (*Nicotiana tabacum* ‘Xanthi’) transgenic plants overexpressing *Ros1* (T_2_-*Ros1-1*, T_2_-*Ros1-2*, T_2_-*Ros1-3*, and T_2_-*Ros1-4*) promoted accumulation of anthocyanin in leaves and flowers by elevating the transcription of all key genes involved in the biosynthesis of this pigment. This promotion largely occurred through the upregulation of dihydroflavonol 4-reductase (*DFR*), and anthocyanidin synthase genes in leaves and upregulation of *DFR* in flowers. Under normal conditions, the transgenic lines and wild type (WT) plants showed well-developed broad leaves and regular roots, whereas a reduction in plant growth was observed under cold and drought stresses. However, the transgenic T_2_-*Ros1* lines were able to tolerate the stresses better than the WT line by inducing reactive oxygen species scavenging activities, and the expression of antioxidant-related and stress-responsive genes. In addition, phylogenetic analysis clustered Ros1 with many transcription factors (TFs) that confer tolerance to different abiotic stresses. Overall, the results obtained here suggest that *Ros1* overexpression upregulates anthocyanin biosynthetic, antioxidant-related, and stress-responsive genes thereby enhancing anthocyanin accumulation and abiotic stress tolerance.

## Introduction

Abiotic stresses such as temperature, soil salinity, light, and drought adversely affect crop productivity and quality by arresting plant growth and development. A better understanding of stress responses is critical for agricultural and economic performance as it might contribute to improve crop tolerance to such abiotic stresses (Varshney et al., 2012). Reactive oxygen species (ROS) play an important role as signaling molecules that initiate stress responses in plants. However, excess accumulation of ROS in the vacuoles disturbs cellular homeostasis, ultimately leading to death by cytotoxicity (Kim et al., 2008). Cold stress, for instance, enhances the production of ROS such as hydrogen peroxide (H_2_O_2_) in the vacuole, which disrupts the electron transport chain, damages cellular processes, and alters plant physiology (Gill and Tuteja, 2010); these changes result in the reduction of crop yield. Similarly, drought stress induces ROS production, which ultimately ceases plant cell division and expansion rate, thus negatively affecting plant growth (Farooq et al., 2009). These reactions eventually lead to crop biomass reduction.

Anthocyanins are flavonoid pigments synthesized via the three main branches of the anthocyanin biosynthetic pathway in plants (**Supplementary Figure 1**). The transcriptional regulation of anthocyanin biosynthesis genes, such as chalcone synthase (*CHS*), chalcone isomerase (*CHI*), flavanone 3-hydroxylase (*F3H*), flavonoid 3′-hydroxylase (*F3′H*), dihydroflavonol 4-reductase (*DFR*), and anthocyanidin synthase (*ANS*), are involved in the three main branches and lead to the production of different flavonoid pigments (ranging from red to blue) in many plant species. The R2R3- myeloblastosis (MYB) transcription factors (TFs) have been shown to enhance anthocyanin production (Hichri et al., 2011) by regulating the genes involved in the anthocyanin biosynthetic pathway in many plants, including model and horticultural crops (Albert et al., 2014; Xie et al., 2014; Zhang et al., 2017). Previous studies have demonstrated that many genes encoding enzymes involved in antioxidant production and abiotic stress tolerance were activated in plants exposed to cold and drought stress conditions (Guy et al., 1994; Thomashow, 1999; Xie et al., 2018), and some R2R3-MYBs are involved in the enhancement of abiotic stress tolerance (Cheng et al., 2013; Meng et al., 2014; Qi et al., 2015). For example, the overexpression of the MYB TF Anthocyanin 2 in *Lycopersicum esculentum* (LeAN2) increased anthocyanin accumulation and enhanced chilling and oxidative stress tolerance in this crop (Meng et al., 2014). Similarly, overexpression of SbMYB7 and SbMYB8 in *Scutellaria baicalensis* induced phenylpropanoid accumulation and enhanced oxidative stress resistance in transgenic tobacco (Qi et al., 2015). Moreover, IbMYB1 from *Ipomoea batatas* positively regulates anthocyanin and salinity tolerance in transgenic potato (Cheng et al., 2013). Although numerous MYB TFs have been identified as anthocyanin regulators, only few have been shown to be involved in anthocyanin regulation and stress tolerance (Cheng et al., 2013; Qi et al., 2015; Yuan et al., 2015).

Rosea1 (Ros1) induces strong red coloration in snapdragon flowers by activating the transcript levels of *F3H, F3′H*, and *DFR* (Schwinn et al., 2006). In addition, overexpression of *Ros1* in lisianthus also promoted pigmentation in its flowers by activating the transcript levels of *CHS* and *ANS* (Schwinn et al., 2006), and the overexpression of *Ros1* with genes *Delila* (*Del*) or *Lc* in tomato and petunia also resulted in higher pigmentation in tomato fruits and petunia flowers by regulating the transcript levels of anthocyanin biosynthesis genes (Butelli et al., 2008; Schwinn et al., 2014). However, previous studies have not examined the role of *Ros1* beyond anthocyanin regulation. In addition, pigmentation was limited in the vegetative tissues of the studied crops. Recently, Naing et al. (2017) reported that the overexpression of *Del* in tobacco not only enhanced anthocyanin accumulation but also improved abiotic stress tolerance. Hence, it would be interesting to further elucidate the role of *Ros1* in anthocyanin regulation and examine if it is also involved in abiotic stress tolerance.

Because anthocyanins contain high contents of enzymatic and non-enzymatic antioxidants that can scavenge excess ROS (Gill and Tuteja, 2010) and, generally, anthocyanins’ accumulation in the vacuoles prevents the overproduction of ROS due to abiotic stresses (Hernandez et al., 2009). In the present study, we generated transgenic tobacco overexpressing snapdragon-derived *Ros1*, and investigated its role in anthocyanin production in vegetative and floral tissues. In addition, we examined the involvement of *Ros1* in the mechanisms that control cold and drought tolerance by determining plant growth parameters, biochemical and physiological changes, stress-induced antioxidant activities, and relevant genes’ expression.

## Materials and Methods

### Plasmid Construction

*Agrobacterium tumefaciens* strain AGL1 harboring the binary vector pJAM1980, which contains the *Antirrhinum majus* *Ros1* gene, was provided by Prof. Cathie Martin (John Innes Centre, Norwich, United Kingdom). The *Ros1* cDNA was placed under the control of the cauliflower mosaic virus (CaMV) 35S promoter, and the neomycin phosphotransferase gene (*nptII*), which confers kanamycin resistance, was used as the selection marker (**Supplementary Figure 2**).

### Genetic Transformation

Leaves of 6-week-old tobacco (*Nicotiana tabacum*, cv. Xanthi) plants grown *in vitro* were cut into 0.5–1.0 cm segments and used as explants for transformation, as described by Naing et al. (2015). Briefly, leaf explants were co-cultivated with the cell suspension of AGL1 carrying pJAM1980 (optical density at 600 nm = 0.5) for 3 days, and then placed on the selection medium [Murashige and Skoog (MS), 1.0 mg L^−1^; 6-benzyladenine, 0.5 mg L^−1^; indole-butyl-acetic acid, 30 g L^−1^; sucrose, 300 mg L^−1^; cefotaxime; and kanamycin, 50 mg L^−1^) for 4 weeks. Red-colored shoots among those emerged from the explants cultured on the selection medium were then rooted on MS medium containing 30 g L^−1^ sucrose, 100 mg L^−1^ cefotaxime, and 50 mg L^−1^ kanamycin and acclimatized in a greenhouse.

### Production of Transgenic T_2_ Progeny

To produce transgenic T_2_-*Ros1* progeny, the transgenic lines obtained from the explants (i.e., T_0_-*Ros1-1*, T_0_-*Ros1-2*, T_0_-*Ros1-3*, and T_0_-*Ros1-4*) showing similar anthocyanin phenotype were grown in a greenhouse and self-pollinated to produce T_1_ seeds. These were grown as the previous generation and the resulting T_1_ seed pods were further self-pollinated for T_2_ seed production. The T_2_ seeds were germinated on peat-based soil, and red-colored shoots (T_2_) were selected by visual screening. Seeds of the different T_2_-*Ros1* lines (T_2_-*Ros1-1*, T_2_-*Ros1-2*, T_2_-*Ros1-3*, and T_2_-*Ros1-4*) obtained by self-pollination were used for further experiments.

### Analysis of Anthocyanin Content

To analyze the total anthocyanin levels accumulated in T_2_-*Ros1* (T_2_-*Ros1-1*, T_2_-*Ros1-2*, T_2_-*Ros1-3*, and T_2_-*Ros1-4*) and wild type (WT) plants, leaves and flowers (approximately 500 mg) were excised from transgenic and WT lines grown in the greenhouse and crushed in liquid nitrogen. Pigments were extracted with 5 mL of 1% (w/v) hydrochloric acid in a methanol solution, as described by Naing et al. (2015), and incubated overnight at 4°C. After centrifugation at 3000 rpm for 20 min, the obtained supernatant was transferred to a 2-mL collection tube and its absorbance was measured at 430–630 nm (spectrophotometer U-2800: Hitachi, Tokyo, Japan). Five biological samples (leaves and flowers) per line (T_2_-*Ros1-1*, T_2_-*Ros1-2*, T_2_-*Ros1-3*, T_2_-*Ros1-4*, and WT) were used, and analysis was performed in triplicate.

### RNA Extraction and Expression Analysis of Anthocyanin Biosynthesis Genes

To analyze the transcript levels of anthocyanin biosynthesis genes (*NtCHI, NtCHS, NtF3H, NtDFR*, and *NtANS*) expressed in WT and T_2_-*Ros1* (T_2_-*Ros1-1*, T_2_-*Ros1-2*, T_2_-*Ros1-3*, and T_2_-*Ros1-4*) lines by quantitative real-time PCR (qRT-PCR), total RNA was isolated from 100 mg of leaf and floral tissues using TRI Reagent^TM^ Solution (Ambion, Thermo Fisher Scientific, Waltham, MA, United States). Following the manufacturer’s protocol, 1 μg of total RNA and an oligo dT_20_ primer were used for reverse transcription with ReverTra Ace^®^ (Toyobo, Osaka, Japan). Transcript levels of *NtCHI, NtCHS, NtF3H, NtDFR*, and *NtANS* were measured using a StepOnePlus Real-Time PCR system (Thermo Fisher Scientific) as described by Naing et al. (2017). Primers and PCR conditions used for detection of the structural genes and the reference gene *NtActin* are listed in **Supplementary Table 1**. Three biological samples (leaves and flowers) per line (T_2_-*Ros1-1*, T_2_-*Ros1-2*, T_2_-*Ros1-3*, T_2_-*Ros1-4*, and WT) were used, and analysis was performed in triplicate.

### Phylogenetic Analysis of Ros1

To determine the potential involvement of *Ros1* in the tolerance to different abiotic stresses, amino acid sequences of Ros1 were aligned with those of 35 MYBs involved in tolerance to cold, salt, drought, and heavy metal stresses in several plant species. The obtained alignments were used to construct a maximum likelihood phylogenetic tree. Initial tree(s) for the heuristic search were obtained automatically by applying neighbor-joining (NJ) and BioNJ algorithms to a matrix of pairwise distances estimated using a JTT model. All analyses were performed in MEGA7.

### Abiotic Stress Experiment

#### Plant Materials

Seeds of transgenic T_2_-*Ros1* (T_2_-*Ros1-1*, T_2_-*Ros1-2*, T_2_-*Ros1-3*, and T_2_-*Ros1-4*) and WT tobacco plants were sown on seedling beds in a greenhouse with an average temperature of 22°C. Seedlings were allowed to grow for 2 weeks, and transgenic T_2_-*Ros1* and WT plants with similar anthocyanin phenotypes were then transferred to individual pots containing peat-based soil for the next 4 weeks. After this period, 6-week-old uniform and healthy transgenic T_2_-*Ros1* and WT plants were used for abiotic stress experiments.

#### Cold Stress Treatment

Six-week-old transgenic T_2_-*Ros1* (T_2_-*Ros1-1*, T_2_-*Ros1-2*, T_2_-*Ros1-3*, and T_2_-*Ros1-4*) and WT plants (30 plants per line) grown in the greenhouse were subjected to cold treatment in a growth chamber set to 0°C; another set of transgenic T_2_-*Ros1* and WT plants were placed in a growth chamber set at 22°C and used as controls. Growth chambers where the plants were maintained were set to ∼60% relative humidity and 16-h photoperiod. Growth variables (plant height and fresh weight) were measured in cold-treated and control plants at the end of treatment (30 days) and compared: This experiment was repeated three times.

#### Drought Stress Treatment

Six-week-old transgenic T_2_-*Ros1* (T_2_-*Ros1-1*, T_2_-*Ros1-2*, T_2_-*Ros1-3*, and T_2_-*Ros1-4*) and WT plants (30 plants per line) grown under irrigated conditions were subjected to drought stress (without watering) for 30 days; another set of transgenic T_2_-*Ros1* and WT plants were maintained in irrigated conditions and used as controls. After 30 days of treatment, the drought-treated plants were re-watered as usual, and growth variables were measured in treated and non-treated plants and compared. This experiment was repeated three times.

#### Determination of ROS Scavenging Activity

To determine ROS scavenging activity, leaves of WT and transgenic T_2_-*Ros1* plants subjected to cold stress and grown under normal conditions (control) were collected 21 days after the onset of the experiment. The scavenging activity of ROS, namely hydroxyl radicals, superoxide radicals, and H_2_O_2_ was measured using 2,2-diphenyl-1-picrylhydrazyl (DPPH) and 2,2′-azino-bis (ABTS) assays (Kim et al., 2014). Five biological samples (leaves) per line (T_2_-*Ros1-1*, T_2_-*Ros1-2*, T_2_-*Ros1-3*, T_2_-*Ros1-4*, and WT) were used, and analyses were performed in triplicate.

#### RNA Extraction and Gene Expression Analysis

To determine the transcript levels of the genes coding for antioxidant enzymes [catalase (CAT), superoxide dismutase (SOD), and peroxidase (POX)] in non-treated (control) and cold and drought stress-treated WT and transgenic T_2_-*Ros1* plants, a qRT-PCR analysis was performed. The transcript levels of abiotic stress-related genes [CRTDRE-binding Factor 1 (*CBF1*) and *Osmotin*], and abscisic acid responsive element-binding factor (*ABF*) were also evaluated in plants subject to the cold stress experiment. Leaf tissues of plants from the cold stress experiment were sampled 21 days after the onset of the experiment, while leaf tissues of plants from the drought stress experiment were sampled 15 days after the onset of the experiment. Total RNA extraction and analysis of gene expression by qRT-PCR were performed as described for anthocyanin biosynthesis genes. The primers and PCR conditions are listed in **Supplementary Tables 2**, **3**. Three biological samples (leaves) per line (T_2_-*Ros1-1*, T_2_-*Ros1-2*, T_2_-*Ros1-3*, T_2_-*Ros1-4*, and WT) were used, and analyses were performed in triplicate.

#### Determination of Stomatal Density, Relative Water Content (RWC), and Malondialdehyde (MDA) Content

To analyze leaf stomatal density, RWC, and MDA, the fourth leaves from the top of WT and transgenic T_2_-*Ros1* plants subjected to drought stress and their respective controls were collected 15 days after the onset of the experiment. Stomatal density was determined following the method of Chung et al. (2014). To determine RWC, fresh leaf weight was recorded immediately after sampling. Leaves were then floated in deionized water at 4°C overnight, before recording their rehydrated weights, and oven-dried at 70°C overnight, to record dry leaf weight. The formula for determining RWC was: RWC = (fresh weight - dry weight)/(rehydrated weight - dry weight). The MDA content was determined in the same leaves, following the method of Bates et al. (1973). Five biological samples (leaves) per line (T_2_-*Ros1-1*, T_2_-*Ros1-2*, T_2_-*Ros1-3*, T_2_-*Ros1-4*, and WT) were used, and analyses were performed in triplicate.

### Statistical Analyses

Data were statistically analyzed using SPSS version 11.09 (IBM Corporation, Armonk, NY, United States) and are presented as means ± standard errors (SE). Duncan’s multiple range test was used to evaluate differences between means, setting significance at *P* < 0.05.

## Results

### Production of Transgenic Lines Expressing *Ros1* and Their T_2_ Generation

Tobacco leaf segments transformed with the *Ros1* gene construct evidenced a callus on the regeneration medium containing kanamycin after 2 weeks of culture, and emergence of shoots from the callus started on the same medium after 4 weeks of culture. Most emerged shoots were red in color, indicating high accumulation of anthocyanin, which remained in the shoots until they were transferred to the MS basal medium for plant growth and rooting. Because the shoots exhibited distinct red coloration, it was easy to select *Ros1*-inserted transgenic shoots without performing a PCR.

**FIGURE 1:**
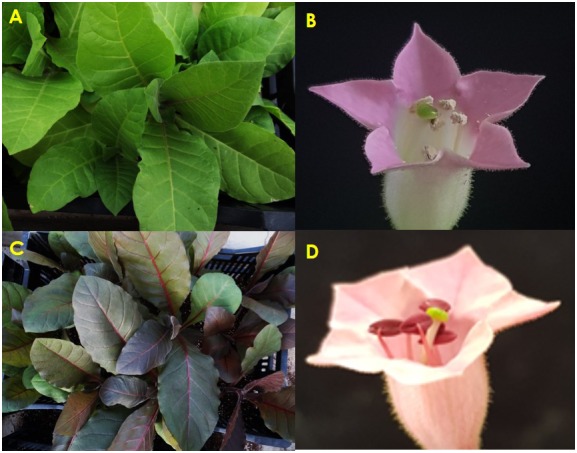
Comparing the difference in phenotypes between WT and T_0_-Ros1 plants; leaves of WT **(A)** and T_0_-Ros1 **(C)**, and flowers of WT **(B)** and T_0_-Ros1 **(D)** (under greenhouse conditions).

Under greenhouse conditions, pigmentation was still observed in whole transgenic T_0_-*Ros1* plants, whereas WT plants displayed green color (**Figures 1A,C**). Upon flowering, red pigmentation was seen in petals or corolla tubes, filaments, and anthers of transgenic flowers but not observed in the floral organs of WT flowers (**Figures 1B,D**). Based on phenotypes, the four transgenic lines (T_0_-*Ros1-1*, T_0_-*Ros1-2*, T_0_-*Ros1-3*, and T_0_-*Ros1-4*), which showed similar and stable pigmentation phenotype, were selected for further experiments.

Most (approximately 75%) of transgenic T_2_ seedlings (T_2_-*Ros1-1*, T_2_-*Ros1-2*, T_2_-*Ros1-3*, and T_2_-*Ros1-4*) obtained by successive self-pollination were red in color, while the remaining were green (data not shown), in agreement with Mendelian inheritance ratio (3:1).

### Anthocyanin Levels in WT and Transgenic T_2_-*Ros1* Plants

**FIGURE 2:**
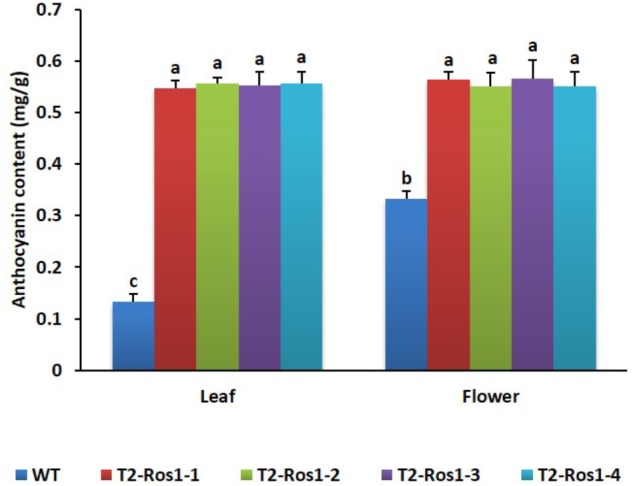
Comparative analysis of anthocyanin content in leaves and flowers of WT and four independent T_2_-*Ros1* lines. Error bars indicate the standard errors (SE) of means of three replicates. Means with different letters are significantly different (DMRT, *p* < 0.05).

The accumulation of anthocyanin in transgenic T_2_-*Ros1* (T_2_-*Ros1-1* and T_2_-*Ros1-2*) plants was significantly higher than in WT plants. The average anthocyanin content detected in leaves of WT, T_2_-*Ros1-1*, T_2_-*Ros1-2*, T_2_-*Ros1-3*, and T_2_-*Ros1-4* plants was 0.13, 0.55, 0.56, 0.55, and 0.56 mg g^−1^ fresh weight, respectively, and that detected in their flowers was 0.33, 0.56, 0.55, 0.55, and 0.55 mg g^−1^ fresh weight, respectively (**Figure 2**).

### Expression of Anthocyanin Biosynthesis Genes in WT and Transgenic T_2_-*Ros1* Plants

**FIGURE 3:**
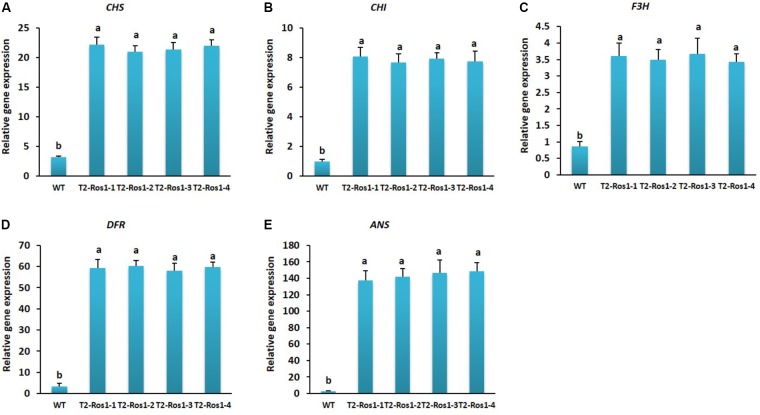
Transcriptional analysis of anthocyanin biosynthetic gene in leaves of WT and four independent T_2_-*Ros1* lines **(A–E)**. *NtActin* gene was used as reference gene to normalize the transcript levels of the genes. Error bars indicate the standard errors (SE) of means of three replicates. Means with different letters are significantly different (DMRT, *p* < 0.05).

**FIGURE 4:**
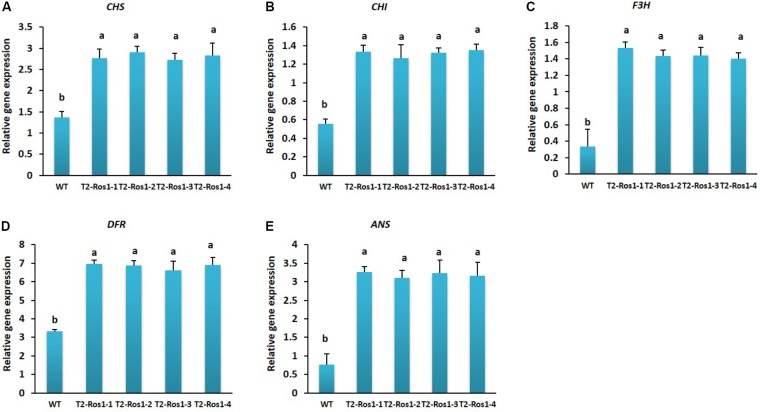
Transcriptional analysis of anthocyanin biosynthetic gene in flowers of WT and four independent T_2_-*Ros1* lines **(A–E)**. *NtActin* gene was used as reference gene to normalize the transcript levels of the genes. Error bars indicate the SE of means of three replicates. Means with different letters are significantly different (DMRT, *p* < 0.05).

Transcript levels of anthocyanin biosynthesis genes were significantly higher in the leaves of transgenic T_2_-*Ros1* plants than in that of WT plants, but not significantly different among the four transgenic lines (T_2_-*Ros1-1*, T_2_-*Ros1-2*, T_2_-*Ros1-3*, and T_2_-*Ros1-4*), in agreement with the different pigmentation observed between WT and transgenic plants (**Figures 3A–E**). Notably, transcript levels of *DFR* and *ANS* were extremely high in transgenic T_2_-*Ros1* lines. All genes analyzed could also be detected in both transgenic and WT flowers. However, as observed for leaves, transcript levels of all genes were higher in transgenic T_2_-*Ros1* flowers than in WT flowers (**Figures 4A–E**), and the higher upregulation of this genes in T_2_-*Ros1* plants was associated with the redder coloration in their flowers than in WT flowers. However, *DFR* was the most highly expressed gene in both transgenic and WT flowers. Overall, overexpression of *Ros1* could upregulate the transcript levels of all anthocyanin biosynthesis genes in vegetative and floral tissues, and these enhanced transcriptional levels contributed to the increased red pigmentation in vegetative and floral tissues.

### Phylogenetic Analysis of Ros1 and Other MYBs Conferring Abiotic Stress Tolerance

**FIGURE 5:**
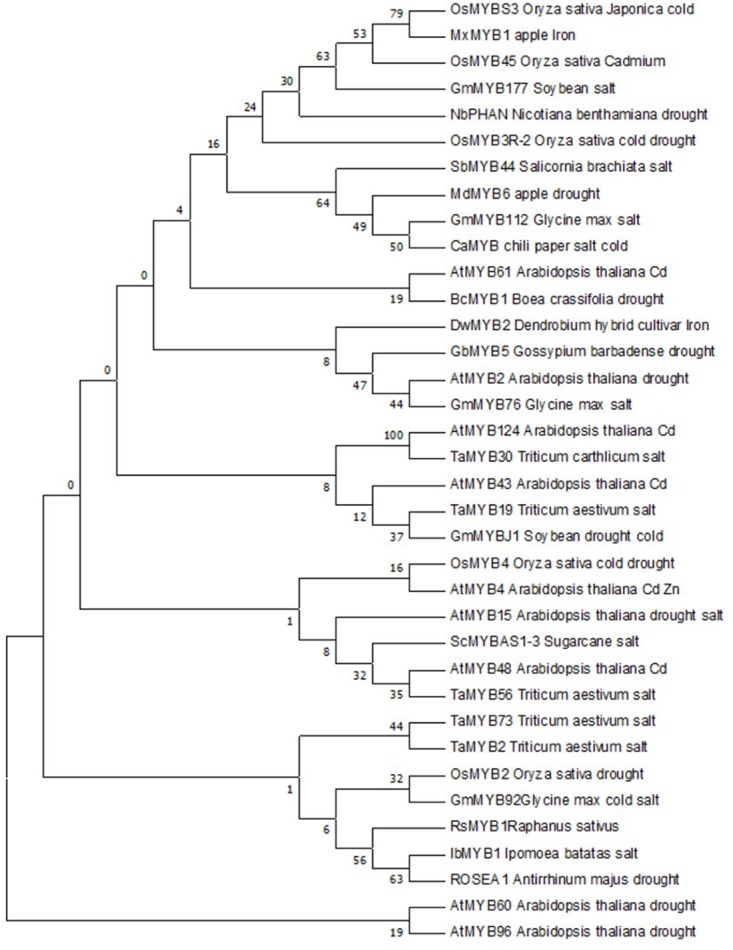
Phylogenetic relationships of the Ros1 MYB with other 35 MYBs that confer different abiotic stress tolerances. The evolutionary history was inferred using the Maximum Parsimony method. Tree #1 out of 3 most parsimonious trees (length = 943) is shown. The consistency index is (0.538298), the retention index is (0.371014), and the composite index is 0.200261 (0.199716) for all sites and parsimony-informative sites (in parentheses). The percentage of replicate trees in which the associated taxa clustered together in the bootstrap test (1500 replicates) are shown next to the branches (Felsenstein, 1985). The MP tree was obtained using the Subtree-Pruning-Regrafting (SPR) algorithm [pg. 126 in ref. (Nei and Kumar, 2000)] with search level 1 in which the initial trees were obtained by the random addition of sequences (10 replicates). The analysis involved 35 amino acid sequences. All positions containing gaps and missing data were eliminated. There were a total of 59 positions in the final dataset. Evolutionary analyses were conducted in MEGA7 (Kumar et al., 2016).

We clarified the potential involvement of Ros1 in abiotic stress tolerance analyzing its phylogenetic relationships with 35 MYB TFs conferring tolerance to cold, drought, salt, and heavy metal stresses in several plant species. According to phylogenetic tree (**Figure 5**), Ros1 was clustered with four MYBs (IbMYB1, GmMYB92, OsMYB2, and RsMYB1) that confer different abiotic stress tolerances to various crops.

### Different Tolerance of the Plants to Cold and Drought Stresses

**FIGURE 6:**
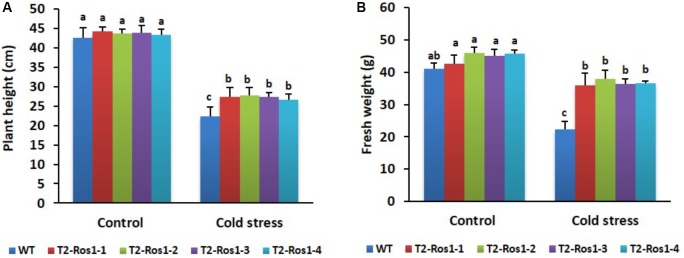
Comparisons of **(A)** plant height and **(B)** fresh weight in 10-week-old WT and four independent T_2_-*Ros1* lines under cold stress (0°C) and normal growth conditions (22°C). Error bars indicate the standard errors (SE) of means of three replicates. Means with different letters are significantly different (DMRT, *p* < 0.05).

**FIGURE 7:**
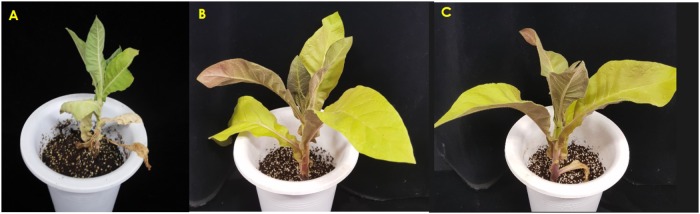
Comparisons of the degree of cold stress tolerance between 10-week-old WT **(A)** and independent T_2_-*Ros1* lines **(B,C)** under the cold stress. Photos were taken on the 7th day after removal from cold stress condition (0°C) to the normal growing condition (22°C).

After being subjected to cold stress conditions for a few days, the transgenic T_2_-*Ros1* plants slightly recovered from the immediate cold stress, whereas WT plants did not. Generally, the growth of WT and transgenic T_2_-*Ros1* plants decreased under cold stress conditions in relation to that of plants grown under normal greenhouse conditions (control). After 21 days of treatment, it was observed that plant height and fresh weight were more influenced by cold stress in WT than in transgenic T_2_-*Ros1* plants, and there were not significant differences among the transgenic T_2_-*Ros1* lines (**Figures 6A,B**). In fact, only the upper two or three leaves remained green in WT plants, and those leaves were found to be smaller, thicker, and more stressed than the leaves of transgenic T_2_-*Ros1* plants. At the end of the cold stress treatment, when the treated plants were transferred to the greenhouse for 7 days the transgenic T_2_-*Ros1* plants totally recovered from the cold stress and grew as normal healthy plants, whereas the WT plants did not recover and remained unhealthy in appearance (**Figure 7**), indicating that the presence of *Ros1* could distinctly enhance tobacco cold tolerance.

**FIGURE 8:**
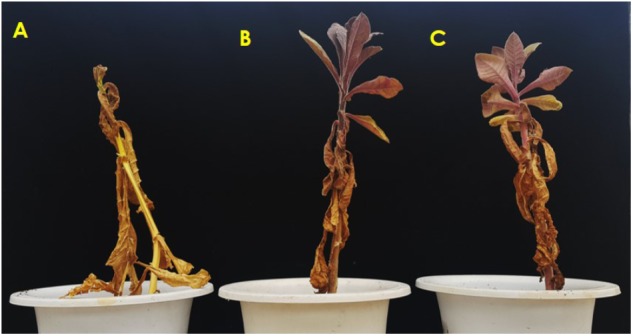
Comparison of the tolerance of the drought stress in WT **(A)** and independent T_2_-*Ros1* lines **(B,C)** under drought stress. Induction of drought stress was observed in the plants after 20 days of the drought stress, and the plants were not watered for 30 days. Following the stress, the plants were regularly re-watered, and the photos were taken 10 days after re-watering.

Under normal growing conditions (control), both WT and transgenic T_2_-*Ros1* plants survived well and their growth parameters, such as leaf size and plant height did not substantially differ (data not shown). However, some leaves began to wilt when the plants were not watered for 7 days. Almost all leaves of the WT plants became yellow after 20 days of drought; in contrast, only the lower leaves of transgenic T_2_-*Ros1* plants appeared yellow while the upper leaves remained green. When plants were not watered for 30 days, all leaves and stems of the WT plants dried and dropped; in transgenic T_2_-*Ros1* plants, only lower leaves dried and dropped whereas upper leaves appeared to only wilt, without stem breakage. To investigate their recovery from drought stress, plants were re-watered 30 days after drought; while no WT plants recovered from drought stress until 10 days after re-watering, new shoots were visible in T_2_-*Ros1* plants after n days (**Figure 8**). Therefore, as observed for cold stress, overexpression of *Ros1* was able to distinctly enhance tobacco drought tolerance.

### Analysis of ROS Scavenging Activity in WT and Transgenic T_2_-*Ros1* Plants

**FIGURE 9:**
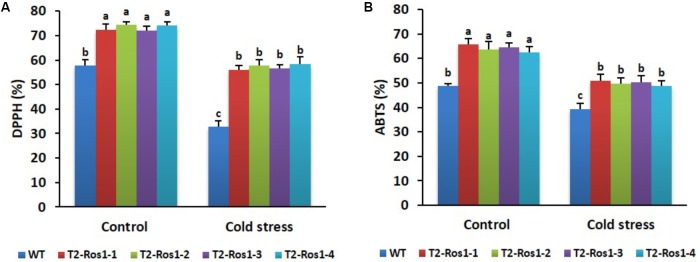
Comparison of ROS scavenging activities between 9-week-old WT and four independent T_2_-*Ros1* lines under cold stress and normal growth conditions; **(A)** DPPH and **(B)** ABTS activities. Data were taken on the 21st day after starting the experiments. Error bars indicate the SE of means of three replicates. Means with different letters are significantly different (DMRT, *p* < 0.05).

Scavenging activities were significantly lower in stress-treated plants than in non-treated plants. Although the ROS scavenging was not significantly different among transgenic T_2_-*Ros1* lines, they were higher than in WT plants, under both stress conditions, but especially under cold-stress conditions (**Figures 9A,B**). These results suggest a higher degree of cold stress tolerance in transgenic T_2_-*Ros1* plants than in WT plants.

### Expression Pattern of Antioxidant-Related Genes

**FIGURE 10:**
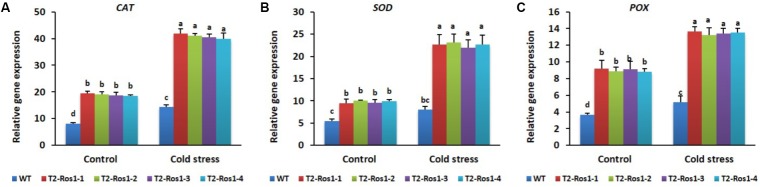
Expression analysis of the antioxidant-related genes (**A**, *CAT*; **B**, *SOD*; **C**, *POX*) in the 9-week-old WT and four independent T_2_-*Ros1* lines under cold stress and normal growth conditions. Data were taken on the 21st day after starting the experiments. *NtActin* gene was used as reference gene to normalize the transcript levels of the genes. Error bars indicate the SE of means of three replicates. Means with different letters are significantly different (DMRT, *p* < 0.05).

Transcript levels of antioxidant-related genes were significantly higher in the transgenic T_2_-*Ros1* plants than in WT plants under control conditions, and increased under stress conditions, although not differing significantly among transgenic T_2_-*Ros1* lines (**Figures 10A–C**). Thus, the higher cold-stress tolerance in transgenic T_2_-*Ros1* than in WT plants seemed to be directly related to the transcript levels of antioxidant-related genes, as the overexpression of *Ros1* strongly induced the expression of antioxidant-related genes under stress conditions. These results also support the hypothesis that the higher cold-stress tolerance in transgenic T_2_-*Ros1* plants depends on their antioxidant content.

**FIGURE 11:**
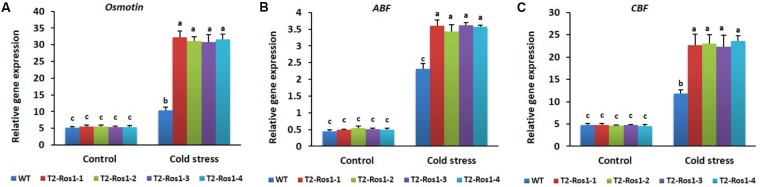
Expression analysis of cold stress-responsive genes (**A**, *Osmotin*; **B**, *ABF*; **C**, *CBF*) in the 9-week-old WT and four independent T_2_-*Ros1* lines under cold stress and normal growth conditions. Data were taken on the 21st day after starting the experiments. *NtActin* gene was used as reference gene to normalize the transcript levels of the genes. Error bars indicate the SE of means of three replicates. Means with different letters are significantly different (DMRT, *p* < 0.05).

Expression patterns of the abiotic stress-related genes *CBF, Osmotin*, and *ABF* were similar to those of antioxidant genes (*SOD, CAT*, and *POX*). Their expression patterns between WT and transgenic T_2_-Ros1 plants were not significantly different under control conditions, but increased gene expression was observed under cold stress conditions, and the induction of these genes in transgenic T_2_-*Ros1* plants was significantly higher than in WT plants (**Figures 11A–C**), which was consistent with the higher degree of cold tolerance in transgenic T_2_-*Ros1* than in WT plants.

**FIGURE 12:**
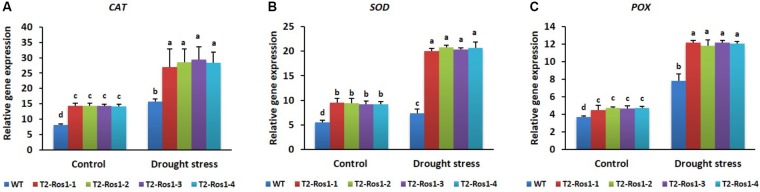
Expression analysis of the antioxidant-related genes (**A**, *CAT*; **B**, *SOD*; **C**, *POX*) in the 8-week-old WT and four independent T_2_-*Ros1* lines under drought stress and normal growth conditions. Data were taken on the 15th day after starting the experiments. *NtActin* gene was used as reference gene to normalize the transcript levels of the genes. Error bars indicate the SE of means of three replicates. Means with different letters are significantly different (DMRT, *p* < 0.05).

Transcript levels of antioxidant-related genes were not significantly different between transgenic T_2_-*Ros1* and WT plants under control conditions; however, under drought stress, transcript levels were significantly higher in transgenic T_2_-*Ros1* plants than in WT plants (**Figures 12A–C**), indicating that *Ros1* promoted drought tolerance by modulating the antioxidant activities that scavenge ROS. Similar to the cold stress condition, no significant differences in transcript levels were found among T_2_-*Ros1* lines under the drought stress condition.

### Analysis of Stomata Density, RWC, and MDA Content

**FIGURE 13:**
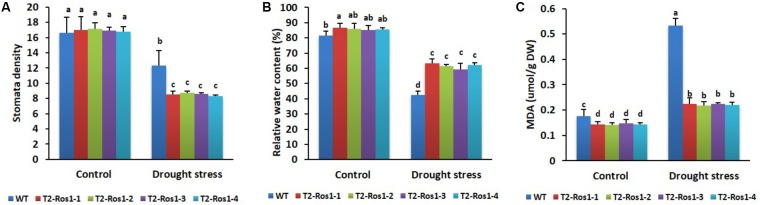
Comparison of leaf stomatal density **(A)**, relative water content **(B)**, and MDA content **(C)** analyzed from the fourth top leaf of 8-week-old WT and four independent T_2_-*Ros1* lines under the drought stress and normal growth conditions. Data were taken on the 15th day after starting the experiments. Error bars indicate the SE of means of three replicates. Means with different letters are significantly different (DMRT, *p* < 0.05).

Leaf stomata density, RWC, and MDA content usually play important roles in drought stress tolerance. Under normal growth conditions (control), these indices were not significantly different between WT and transgenic T_2_-*Ros1* plants. However, under drought stress, stomatal density was significantly higher in WT than in transgenic T_2_-*Ros1* plants (**Figure 13A**), while RWC was significantly lower (**Figure 13B**). These results suggested that reduction of stomatal density caused a reduction in water loss via transpiration, protecting plants against rapid reductions of RWC to increase drought stress tolerance; thus, a high degree of drought tolerance was observed in T_2_-*Ros1* plants. The MDA content was also associated with drought stress tolerance because it was significantly lower in transgenic T_2_-*Ros1* plants than in WT plants (**Figure 13C**), suggesting that the presence of lower MDA content improved the drought stress tolerance of transgenic plants.

## Discussion

Despite the several reports describing a dual role for MYB TFs (anthocyanin production and abiotic stress tolerance) in various plant species, few studies have reported the role of the MYB TF Ros1 in anthocyanin accumulation and in abiotic stress tolerance. Hence, we produced transgenic tobacco plant lines expressing *Ros1* and characterized its dual role in anthocyanin accumulation and abiotic stress tolerance by assessing phenotypic variation, alteration of bio-physiological processes, and gene expression. We found that *Ros1* overexpression in tobacco enhanced anthocyanin accumulation in vegetative (leaves and stems) and floral (petals, corolla, and stamen) tissues, as this accumulation was higher in T_2_-*Ros1* plants than in WT plants. This difference could be explained by the differences in the expression of anthocyanin biosynthesis-related genes in plant tissues, because significantly higher expression of *CHS*, *CHI*, *F3H*, *DFR*, and *ANS* was noted in transgenic T_2_-*Ros1* than in WT plants. However, some genes seemed to play more important roles than others in enhancing anthocyanin production, as the expression levels of *DFR* and *ANS* genes were only slightly detected in WT plants but presented the highest levels in transgenic T_2_-*Ros1* plants. Mooney et al. (1995) demonstrated that the lack of expression of *DFR* was linked to no anthocyanin accumulation in tobacco vegetative tissues. Similarly, Huang et al. (2012) and Naing et al. (2017) reported that the strong induction of *DFR* transcript levels by *Lc* and *Del* led to pigmentation in leaves, whereas in WT no or low expression of *DFR* resulted in the lack of leaf pigmentation. In addition, Naing et al. (2017) reported that *ANS* expression was crucial for the different pigmentation observed in different transgenic lines.

Several researchers successfully modified tobacco flower color via the overexpression of TFs (Nakatsuka et al., 2007; Huang et al., 2012); however, they did not investigate how such TFs regulated endogenous biosynthetic genes, nor did they examine the association between the anthocyanin biosynthetic genes expression and anthocyanin. In the present study, we used qRT-PCR for analyzing the expression of *CHS*, *CHI*, *F3H*, *DFR*, and *ANS* in pink WT and red-pink transgenic T_2_-*Ros1* flowers. Although gene expression was detected in both flower types, the differences in pigmentation were linked to transcriptional variations in anthocyanin biosynthesis genes, with higher gene expression in transgenic than in WT plants. Differences in *DFR* expression was probably the most critical for color differences because of its high transcript levels in both flower types. Mooney et al. (1995) also reported that although *DFR* was absent in the green leaves of the tobacco cultivar ‘Samsam,’ it was detected in its light-pink flowers, and that the upregulation of *DFR* by *Del* overexpression led to enhanced flower pigmentation. In addition, Han et al. (2012) showed that inhibition of *CHI* and *DFR* expression in tobacco flowers decreased anthocyanin accumulation. Recently, Naing et al. (2017) also claimed that *DFR* is likely to be more critical for flower color differences in transgenic lines of the same cultivar. Hence, enhancement of pigmentation in the flowers of transgenic lines relative to that of WT was likely due to the upregulation of *DFR* by *Ros1* overexpression. Taken together, the introduction of *Ros1* into tobacco significantly enhanced its anthocyanin contents in vegetative and flower tissues by upregulating all anthocyanin biosynthetic genes. While *DFR* and *ANS* are likely the most important genes for the red pigmentation of vegetative tissues, *DFR* is the most important for floral pigmentation.

All transgenic lines (T_2_-*Ros1-1*, T_2_-*Ros1-2*, T_2_-*Ros1-3*, and T_2_-*Ros1-4*) contained higher anthocyanin contents than the WT line, which might generate higher ROS scavenging activity in transgenic than in WT line. Thus, it was of interest to investigate the degree of abiotic stress tolerance in transgenic lines relative to that of the WT line. Before abiotic stress experiments, the potential involvement of *Ros1* in abiotic stress tolerance was clarified based on the phylogenetic analysis of Ros1 and 35 MYB TFs known to be related to tolerance to different abiotic stresses. The phylogenetic tree indicated that Ros1 clustered with IbMYB1, GmMYB92, OsMYB2, and RsMYB1 that confer different abiotic stress tolerances in various crops by regulating the expression of stress- and antioxidant-related genes. This suggested that Ros1 function might be similar to that of the four MYBs located in its cluster. In fact, Ros1 sequence was highly similar to that of IbMYB1, which confers anthocyanin accumulation and salt-stress tolerance.

Under normal conditions (control), growth (plant height and fresh weight) of transgenic T_2_-*Ros1* and WT plants was not significantly different. However, significant growth inhibition was observed when plants were exposed to cold stress for 21 days, although this inhibition was more adverse in WT than in transgenic plants throughout the stress period. Inhibition of plant growth under cold stress might be caused by excess generation of ROS, which induce oxidative damage, leading to cell membrane lipid peroxidation and cell injury (Prasad et al., 1994; Mittler, 2002; Fini et al., 2011). This was consistent with the results of ROS scavenging abilities (DPPH and ABTS assays), as ROS scavenging abilities were higher under normal conditions (control) than under cold stress conditions in both WT and transgenic plants. The increased tolerance to cold stress in transgenic T_2_-*Ros1* than in WT plants was probably due to the higher anthocyanin levels in former than in the latter, as higher anthocyanin accumulation has been linked to a greater ROS scavenging ability (DPPH and ABTS) and abiotic stress tolerance (Fini et al., 2011; Nakabayashi et al., 2014). Ahmed et al. (2015) also suggested that induction of anthocyanin biosynthesis by the TFs BrMYB2-2 and BrTT8 increased cold and freezing stress tolerances in *Brassica rapa*. Similarly, enhancement of anthocyanin levels improved cold stress tolerance in sweet potato by activating ROS scavenging activities (Wang et al., 2013). Hence, the present results are consistent with that of previous studies. On the other hand, *Ros1* itself may be directly or indirectly involved in cold stress tolerance, as other MYB TFs (e.g., MdSIMYB1, MdoMYB121, IbMYB1, OsMYB4, and OsMYB2) have been linked to cold stress tolerance in several plant species (Cao et al., 2013; Meng et al., 2014; Wang et al., 2014).

To understand the molecular mechanism of the antioxidant genes *SOD, CAT*, and *POX* that encode the enzymes scavenging ROS, thereby being involved in stress tolerance, the expression levels of these genes were examined in transgenic T_2_-*Ros1* and WT plants under stress and control conditions. The transcript levels induced by cold stress were significantly higher than under normal (control) conditions for both T_2_-*Ros1* and WT plants, and significantly higher in transgenic than in WT plants. The stronger induction of genes under cold stress than under control conditions might be associated with plant response to enhanced ROS formation caused by the cold stress. However, under the same conditions, the higher induction of antioxidant genes in T_2_-*Ros1* than in WT plants was likely due to *Ros1* upregulation of these genes, as observed for the overexpression of other MYB TFs (OsMYB2, MdMYB88, or MdMYB124) (Cao et al., 2013; Meng et al., 2014; Wang et al., 2014). Another explanation is that the presence and high accumulation of anthocyanin in T_2_-*Ros1* are likely linked to high antioxidant activities (Dehghan et al., 2014; Naing et al., 2017). Therefore, the higher cold stress tolerance observed in transgenic T_2_-*Ros1* than in WT plants might be associated with the upregulation of antioxidant-related genes that induce antioxidant activities.

Under cold stress, plants evolved multiple mechanisms by upregulating *CBF*, *Osmotin*, and *ABF* (Fujii et al., 2009; Parkhi et al., 2009; Chinnusamy et al., 2010; Goel et al., 2010; Knight and Knight, 2012; Kim et al., 2016), thereby preventing stress-induced damage. Accordingly, we found significantly higher expression of these genes in plants under cold stress than in plants under control conditions. Moreover, the induction of higher transcript levels in transgenic T_2_-*Ros1* than in WT plants was strongly associated with the degree of cold stress tolerance (T_2_-*Ros1* > WT). Upregulation of these genes in response to cold stress has been reported in previous studies (Choi et al., 2000; Goel et al., 2010; Shi et al., 2014). High induction of *CBF1* expression has been linked to cold stress tolerance (Ito et al., 2006; Wisniewski et al., 2011) and high induction of *Osmotin* expression resulted in the improvement of cold and other abiotic stresses tolerances via accumulation of proline, which is responsible for efficient osmoregulation and ROS scavenging (Goel et al., 2010; Subramanyam et al., 2011). Moreover, strong induction of *ABF* expression by cold conditions (Choi et al., 2000) has been reported to alleviate oxidation damage of cell membranes via the improvement of ROS scavenging (Choi et al., 2000). Furthermore, application of exogenous ABA could enhance plant cold resistance (Kim et al., 2016) by increasing soluble sugar and proline (Huang et al., 2015). Overall, these results suggest that improvement of cold tolerance in transgenic T_2_-*Ros1* plants might, at least partially, be due to the direct regulation of *CBF, Osmotin*, and *ABF* by *Ros1*.

Similar to that observed under cold stress, the growth of transgenic T_2_-*Ros1* and WT plants was delayed under drought stress in comparison with normal (control) conditions, although transgenic plants grew more than WT plants throughout the stress period. In addition, recovery from drought stress by re-watering was only accomplished by transgenic plants, indicating that *Ros1* overexpression enhanced the drought tolerance. This improved tolerance of transgenic plants could be explained by their higher anthocyanin content, as anthocyanin content and drought stress tolerance are directly associated in various plant species (Fini et al., 2011; Zhang et al., 2012; Nakabayashi et al., 2014; Yuan et al., 2015). Another possible explanation might be the upregulation of stress-responsive genes involved in drought stress tolerance by *Ros1*, as the overexpression of some MYB TFs improved drought stress tolerance via this mechanism (Zhang et al., 2012; Su et al., 2014; Chen et al., 2015).

Analysis of stomata density also supported the stress tolerance results, as the lower stomatal density in transgenic T_2_-*Ros1* leaves than in WT leaves might reduce water loss via reducing the transpiration rate. This association between low stomatal density and improved drought stress tolerance is consistent with that reported in previous studies (Wang et al., 2011; Xie et al., 2012). For example, GaMYB85 from cotton was found to confer drought tolerance by reducing stomatal density (Butt et al., 2017). The higher drought stress tolerance of transgenic plants compared to WT plants was further supported by the higher RWC in transgenic T_2_-*Ros1* than in WT plants, which might be attributed to the lower stomatal density in the former than in the latter. However, ROS scavenging by anthocyanin during drought stress thereby maintaining water homeostasis in plants (Nakabayashi et al., 2014) might also be responsible for the higher drought stress tolerance of transgenic plants in relation to WT plants.

As a marker for lipid peroxidation, low MDA content in leaves has been linked to high drought tolerance (Esfandiari et al., 2007; Wang et al., 2016). In agreement, transgenic T_2_-*Ros1* plants showed lower MDA contents than WT plants, in line with their drought stress tolerance. The greater reduction of MDA in transgenic than WT plants was also supported by the transcriptional levels of the antioxidant-related genes *SOD, CAT*, and *POX* (T_2_-*Ros1* > WT), as an increase of antioxidant activities in transgenic plants would reduce MDA contents by scavenging ROS, leading to their enhanced drought tolerance in relation to WT plants. Yang et al. (2012) also claimed that MDA in OsMYB2-overexpressing plants was markedly lower than in WT plants under abiotic stress.

## Conclusion

Overexpression of *Ros1* enhanced anthocyanin accumulation in plants by elevating transcript levels of key genes involved in anthocyanin biosynthesis pathways. In addition, *Ros1* overexpression enhanced tolerance to cold and drought stresses by inducing various alleviation functions, such as alterations in physiological and biochemical processes, transcript regulation of stress-responsive genes, osmotic adjustment, and antioxidant defense. Taken together, the results reported here provide crucial information on the dual role of *Ros1* in anthocyanin accumulation and abiotic stress tolerance, contributing for the elucidation of the mechanism of abiotic stress tolerance by *Ros1*. This gene might be used as a target for genetic manipulation to modify flower color and to improve multiple abiotic stress tolerances for commercially important crops.

## Author Contributions

AN designed the study, conducted the experiment, and wrote the manuscript. CK and IL supervised the experiments at all stages and performed the critical revisions of the manuscript. TA and KL assisted with experimental procedures and data analysis. All authors read and approved the final manuscript.

## Conflict of Interest Statement

The authors declare that the research was conducted in the absence of any commercial or financial relationships that could be construed as a potential conflict of interest.
