# Supplementary material for: Overexpression of Rosea1 From Snapdragon Enhances Anthocyanin Accumulation and Abiotic Stress Tolerance in Transgenic Tobacco
Source: Front Plant Sci. 2018 Aug 15;9:1070. doi: 10.3389/fpls.2018.01070 (PMC6104419; doi:10.3389/fpls.2018.01070)
Supplement: Supplementary file 1 [file Data_Sheet_1.pdf]

Supplementary file

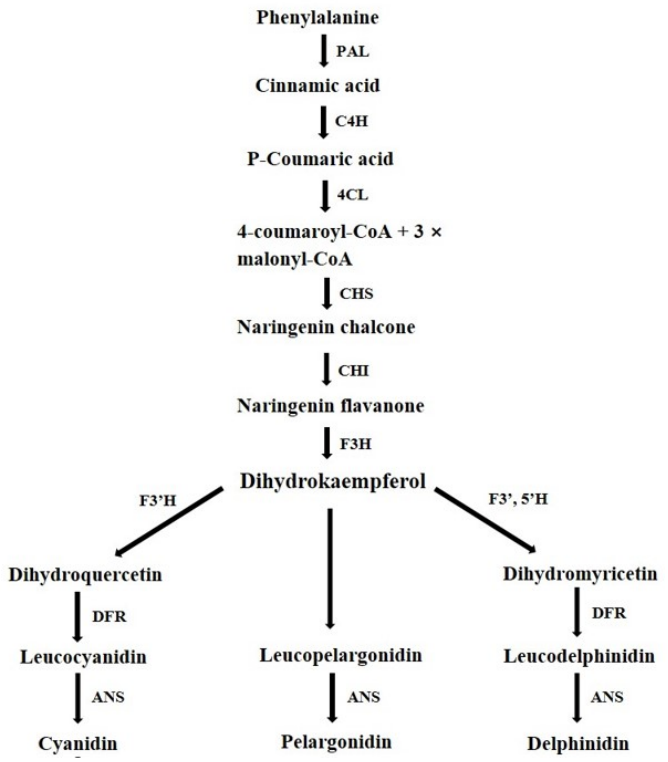

Fig. 1. The schematic diagram of anthocyanin biosynthetic pathway in plants. *CHS*, chalcone synthase enzyme; *CHI*, chalcone isomerase; *F3H*, flavanone 3-hydroxylase; *F3′ H*, flavanone 3′-hydroxylase; *F3′5′H*, flavanone 3′, 5′-hydroxylase; *DFR*, Dihydroflavonol 4-reductase; and *ANS*, anthocyanidin synthase

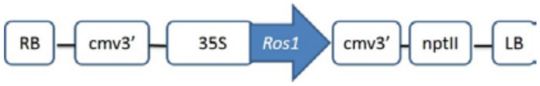

Fig. 2. The T-DNA region of the binary vector pJAM1980 showing the left and right border regions; 35s promoter, the *nptII* gene, and snapdragon *Rosea1* (*Ros1*) cDNA.

Tables

Table 1. Primer sequences for qRT-PCR of anthocyanin structural genes.

Gene	Accession No.	Primer sequences (5′-3′)
CHS	AF311783	F, TTGTTCGAGCTTGTCTCTGC ′
		R, AGCCCAGGAACATCTTTGAG
CHI	AB213651	F, GTCAGGCCATTGAAAAGCTC
		R, CTAATCGTCAATGCCCCAAC
F3H	AB289450	F, CAAGGCATGTGTGGATATGG
		R, TGTGTCGTTTCAGTCCAAGG
DFR	AB289448	F, AACCAACAGTCAGGGGAATG
		R, TTGGACATCGACAGTTCCAG
ANS	AB289447	F, TGGCGTTGAAGCTCATACTG
		R, GGAATTAGGCACACACTTTGC
ACTIN	AB158612	F, GGTCATTACCATTGGCTCAGA
		R, CAACAAGTGATGGCTGGAATAAA

PCR conditions for qRT-PCR: 95°C (10 min) → [95°C (30 s) → 57°C (25 s) →72°C (35 s)] × 40 cycles → 95°C (15 s) → 57°C (30 s) → 95°C (15 s)

Table 2. Primer sequences for qRT-PCR of antioxidant and cold-responsive genes.

Gene	Accession No.	Primer sequences (5′-3′)
SOD	EU342358	F; GCCAGCTTTGAAGATGAACGA<br>R; GCCTAATGCTCTTCCCACCAT
CAT	U93244	F; GATGACAAGATGCTTCAAACTCGTA<br>R; CACTTTGGAGCATTAGCAGGAA
POX	D11396.1	F; ACTGCTCCGTCACCCAAAAC<br>R; GCCCTGGTTCCTGCTTAAGTC
Osmotin	X95308	F; ACTATCGAGGTCCGAAACAACTG<br>R; GCATTGATCACCCAAGTTTGG
ABF	KP050784.1	F; GGCTTGGCCGGATTGC<br>R; TTTCCAGGCGATCCTCGTT
CBF	NM_118679.2	F: CGGTGATTACAGTCCGAAGCTT<br>R: TTCCTTCCCGCTGGTTTCT

Table 3. PCR conditions for qRT-PCR of antioxidant- and proline-related genes.

Genes	PCR conditions:
SOD	95°C (10 min) → [95°C (30 s) → 59°C (30 s)] × 40 cycles → 95°C (15 s) → 59°C (30 s) → 95°C (15 s)
CAT	95°C (10 min) → [95°C (30 s) → 59°C (30 s)] × 40 cycles → 95°C (15 s) → 59°C (30 s) → 95°C (15 s)
POX	95°C (10 min) → [95°C (30 s) → 60°C (30 s)] × 40 cycles → 95°C (15 s) → 60°C (30 s) → 95°C (15 s)
Osmotin	95°C (10 min) → [95°C (30 s) → 59°C (30 s)] × 40 cycles → 95°C (15 s) → 59°C (30 s) → 95°C (15 s)
ABF	95°C (10 min) → [95°C (30 s) → 59°C (30 s)] × 40 cycles → 95°C (15 s) → 59°C (30 s) → 95°C (15 s)
CBF	95°C (10 min) → [95°C (30 s) → 59°C (30 s)] × 40 cycles → 95°C (15 s) → 59°C (30 s) → 95°C (15 s)
